# Prognostic value of changes in resting‐state functional connectivity patterns in cognitive recovery after stroke: A 3T fMRI pilot study

**DOI:** 10.1002/hbm.22439

**Published:** 2014-02-12

**Authors:** R. Dacosta‐Aguayo, M. Graña, A. Savio, M. Fernández‐Andújar, M. Millán, E. López‐Cancio, C. Cáceres, N. Bargalló, C. Garrido, M. Barrios, I. C. Clemente, M. Hernández, J. Munuera, A. Dávalos, T. Auer, M. Mataró

**Affiliations:** ^1^ Department of Psychiatry and Clinical Psychobiology University of Barcelona Spain; ^2^ Department of CCIA Group of Computational Intelligence, University of the Basque Country UPV/EHU San Sebastian Spain; ^3^ Institute for Brain Cognition and Behaviour (IR3C) Barcelona Spain; ^4^ Department of Neurosciences Hospital Germans Trias I Pujol, Universitat Autònoma de Barcelona Badalona Barcelona Spain; ^5^ Diagnostic Center for Image Clinic Hospital Barcelona Spain; ^6^ Imatge Platform of IDIBAPS Barcelona Spain; ^7^ Department of Methodology of Behavioral Sciences University of Barcelona Spain; ^8^ Institut de Diagnostic per la Imatge (IDI) Hospital Germans Trias I Pujol Badalona Barcelona Spain; ^9^ MRC Cognition and Brain Sciences Unit Cambridge United Kingdom

**Keywords:** ischemic stroke, resting state, fMRI, probabilistic independent component analysis, interhemispheric balance, cognitive recovery

## Abstract

Resting‐state studies conducted with stroke patients are scarce. First objective was to explore whether patients with good cognitive recovery showed differences in resting‐state functional patterns of brain activity when compared to patients with poor cognitive recovery. Second objective was to determine whether such patterns were correlated with cognitive performance. Third objective was to assess the existence of prognostic factors for cognitive recovery. Eighteen right‐handed stroke patients and eighteen healthy controls were included in the study. Stroke patients were divided into two groups according to their cognitive improvement observed at three months after stroke. Probabilistic independent component analysis was used to identify resting‐state brain activity patterns. The analysis identified six networks: frontal, fronto‐temporal, default mode network, secondary visual, parietal, and basal ganglia. Stroke patients showed significant decrease in brain activity in parietal and basal ganglia networks and a widespread increase in brain activity in the remaining ones when compared with healthy controls. When analyzed separately, patients with poor cognitive recovery (*n* = 10) showed the same pattern as the whole stroke patient group, while patients with good cognitive recovery (*n* = 8) showed increased activity only in the default mode network and fronto‐temporal network, and decreased activity in the basal ganglia. We observe negative correlations between basal ganglia network activity and performance in Semantic Fluency test and Part A of the Trail Making Test for patients with poor cognitive recovery. A reverse pattern was observed between frontal network activity and the abovementioned tests for the same group. *Hum Brain Mapp 35:3819–3831, 2014*. © **2014 The Authors. Human Brain Mapping published by Wiley Periodicals, Inc.**

## INTRODUCTION

Acute ischemic stroke is the second most common cause of death worldwide and a major cause of disability in the elder population [Gorelick et al., 2011]. Mechanisms underlying functional recovery after stroke have not been clarified so far. Some of the most relevant factors cited are: vascular repair, immunomodulation, endogenous neurogenesis [Bliss et al., 2010; Horie et al., 2011; Liu et al., 2008] and the rewiring of surviving brain circuits enabling the healthy brain to compensate for the loss of functionality corresponding to the damaged area [Benowitz and Carmichael, 2010; Dancause, 2006; Murphy and Corbett, 2009].

Functional imaging and stimulation studies in patients have shown a rewiring of the brain circuits after stroke which, at least in the first few weeks, indicates recruitment of both ipsi‐ and contralesional areas suggesting that this remapping is caused by local and long distant changes in axonal sprouting and dendritic arborization [Gonzalez et al., 2003].

Resting‐state functional magnetic resonance imaging (rs‐fMRI) demonstrates task unrelated brain networks, such as the default mode network (DMN), and networks of functionally related areas, such as the motor, visual, auditory, and attentional networks [Biswal et al., 2010, Buckner et al., 2009]. These resting state networks (RSNs) have shown a high reproducibility across subjects, time and research sites [Damoiseaux et al., 2006], and have been proved as surrogate biomarkers of neurological diseases (including schizophrenia, autism and Alzheimer's disease).

Few resting‐state functional connectivity studies have been conducted with stroke patients so far, and most of them have focused on the study of motor recovery [Carter et al., 2010; Golestani et al., 2012; Park et al., 2011]. These studies have mainly investigated disruptions in interhemispheric resting‐state functional connectivity of attentional and motor networks over a priori selected regions [Calautti et al., 2007; Corbetta et al., 2005; Cramer and Crafton, 2006; Muellbacher et al., 2002]. Therefore, they considered some networks while discarding others that may be equally important for the prognosis of stroke patients. These studies have shown that neuroplasticity occurs, so that focal injury may even result in interhemispheric changes. Some studies suggest that the restoration of perilesional networks, which have escaped irreversible damage, is the principal contribution to recovery, and that the role of the contralesional hemisphere is subsidiary, because it is recruited only when the left hemisphere is severely damaged [Heiss and Thiel, 2006]. However, fMRI studies with language tasks performed very early after the stroke event suggest that activation in the intact right hemisphere is related to the long‐term outcome [Crinion and Leff, 2007].

The study reported in this paper investigates the resting‐state functional connectivity patterns of the whole brain on functional MRI captured three months after a focal stroke event, using the probabilistic independent component analysis (pICA) approach [Beckmann et al., 2005]. pICA does not need a priori definition of a seed region, allowing unbiased exploration of the association between the RSNs and patient's cognitive improvement. Study hypotheses are (1) stroke patients will show changes relative to healthy controls in the RSNs, both in the vicinity of the lesion as well as in remote cortical areas in the injured and healthy hemisphere; (2) one of the RSNs impaired in stroke patients with poor cognitive recovery will be the DMN, because it has already been associated with more successful performance in cognitive tasks [Anticevic et al., 2012], and (3) patients with poor and good cognitive recovery will show different functional connectivity patterns at three months after stroke. To our knowledge, this is the first study describing the functional reorganization of brain activity patterns after stroke in relation to cognitive recovery.

## MATERIALS AND METHODS

### Participants

From September 2010 to May 2012, 26 patients were admitted to the acute stroke unit of the Germans Trias I Pujol University Hospital (Badalona, Spain). Eighteen of them fulfilled the following criteria: (1) Right‐handedness; (2) First focal ischemic stroke in the territories of the anterior, middle, or posterior cerebral arteries (ACA, MCA, PCA, respectively) without significant hemorrhagic transformation; (3) Age between 40 and 75 years; (4) Absence of severe aphasia (fourteenth scoring item of National Institute of Health Stroke Scale (NIHSS) ≤ 1); (5) Absence of alcohol or drug abuse, psychiatric comorbidities, or severe visual or hearing loss; (6) Absence of contraindications to undergo MRI. Eighteen healthy volunteers from the Barcelona Asymptomatic Intracranial Atherosclerosis study [López‐Cancio et al., 2011; Miralbell et al., 2012] matched by age, sex, education, and handedness (Edinburgh Handedness Inventory [Olfield, 1971]) were recruited as the control group. None had a previous history of neurological or psychiatric diseases and brain scans were reported as normal. The study was approved by the ethics committee of the University of Barcelona. All participants received explanation of study procedures and gave their written consent to participate in the study, which was conducted according to the provisions of the Helsinki declaration.

Statistical analyses were performed with the Statistical Package for the Social Sciences (SPSS, Chicago), version 17.0 for Windows. The distributions of demographic variables were tested for normality by the Shapiro‐Wilk test. We assessed group differences using parametric (*t* test) and nonparametric (Mann‐Whitney test) independent sample tests for continuous variables and Chi‐Square or Fisher's exact test for categorical variables. The threshold for two‐sided statistical significance was set at *P* < 0.05.

### Neuropsychological Assessment and Grouping Criteria Regarding Cognitive Recovery

Information about previous cognitive impairment was assessed by a trained neuropsychologist with the short version of the Spanish Informant Questionnaire on Cognitive Decline in the Elderly [Morales‐González et al., 1992] and the Frontal Behavioral Inventory [Kertesz et al., 1997] on admission day. Premorbid Intelligence was estimated using the vocabulary subtest of Wechsler Adults Intelligence Scale (WAIS‐III‐R) [Wechsler, 1999] at three months poststroke. Patients underwent neuropsychological examinations both within 72 h after the stroke (acute phase) and after 3 months (subacute phase). We selected a test battery that covered a variety of possible cognitive manifestations of vascular brain injury. Attentional abilities were explored by the Digit Span Forward Test (WAIS‐III‐R) [Wechsler, 1999], the subtest of attention extracted from the Montreal Cognitive test [Nasreddine et al., 2005], and the Line Cancellation Test [Strauss, 2006]. Executive abilities were assessed with the Digit Span Backwards from WAIS‐III‐R [Wechsler, 1999], part B of Trail Making Test [Strauss, 2006], Phonological fluency (letter P) [Strauss, 2006], and Semantic fluency test (animals) [Strauss, 2006]. Language abilities were assessed listening to patient spontaneous speech (talking briefly about his/her health problems), and with the following tests: the repetition and understanding items extracted from the Mental Status Examination in Neurology [Strub and Black, 2000], the writing one sentence item extracted from the Mini Mental State Examination Test (MMSE) [Folstein, 1983], and the short version (15‐items) of the Boston Naming Test [Kaplan et al., 1983]. Premotor abilities were assessed with Luria's sequences test, Rhythms subtest extracted from the Montreal Cognitive test [Nasreddine et al., 2005], and interference and inhibitory control subtest extracted from the Frontal Assessment Battery [Dubois et al., 2000]. Speed and visuomotor coordination were assessed with the part A of the Trail Making Test [Strauss, 2006] and the grooved pegboard test (GPT) [Ruff and Parker, 1993]. Neuropsychological examinations also included the MMSE [Folstein, 1983), as a global cognitive test and the Geriatric Depression Scale [Yesavage et al., 1982].

The neuropsychological examination at the acute phase was time‐bound to 60 min. If the patient was fatigued, a pause was introduced. The second subacute cognitive examination lasted about 2 h. We only considered the scores of tests included in both examinations. Healthy controls received the same neuropsychological assessment as patients at the acute phase.

Stroke patients were split into two groups according to their level of cognitive recovery between acute and subacute phase by the following process. First, a paired t‐test was conducted over the cognitive test scores to select the tests with overall significant patient improvement. Second, a subject was categorized as a good cognitive recovery patient if he/she had achieved a minimum improvement of 1.5SD of the scores in at least three of the selected tests. Some patients achieve cognitive normalization.

### Lesion Analysis

**Figure 1:**
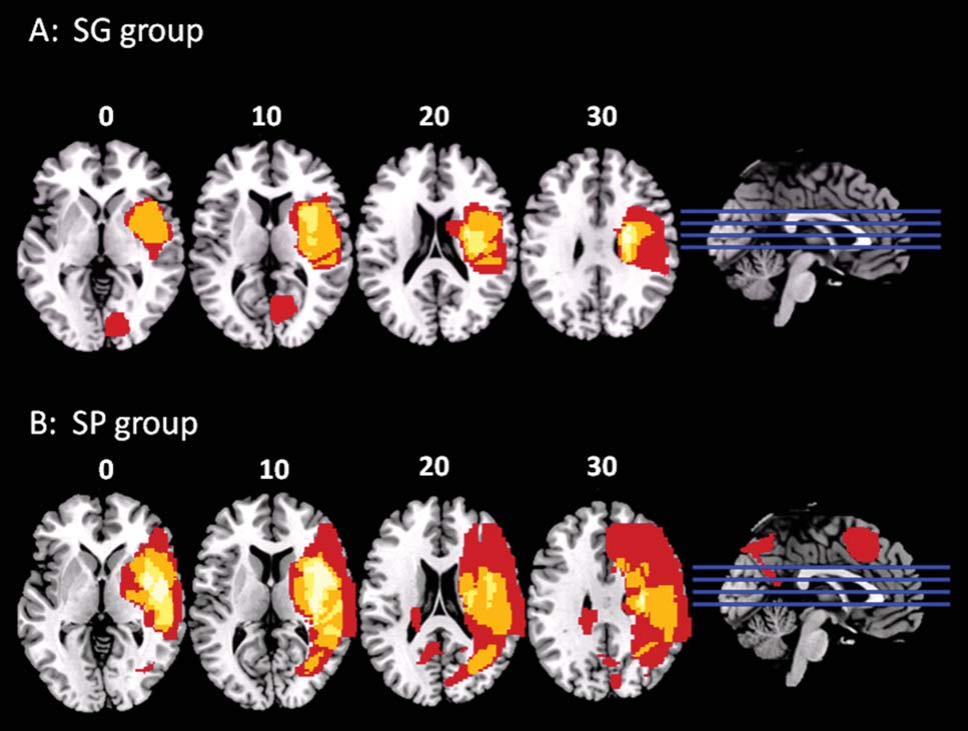
A: Frequency distribution of the lesions for patients with good cognitive recovery. B: Frequency distribution of the lesions for patients with poor cognitive recovery. Images are depicted in radiological convention (R‐L).

Infarct depth (cortical, subcortical or both), laterality (left/right), and vascular territory involved were determined within the first 24 h employing computed tomography and/or magnetic resonance (MRI). Lesion volume was calculated in the subacute phase as the product of the three largest lesion diameters, along the three orthogonal axes, divided by 2 [Sims et al., 2009]. Maps of the lesion distribution for each stroke group are shown in Figure 1.

### Image Analysis

#### fMRI acquisition

fMRI data were acquired in the subacute phase using a Siemens Magneto TIM Trio operating at 3 Tesla at the Image Platform of IDIBAPS, Centre de diagnostic per la Imatge from Hospital Clínic, Barcelona. We used a 32‐channel phased‐array head coil with foam padding and head phones to restrict head motion and scanner noise. Resting‐state blood oxygen level‐dependent data were acquired using an echo‐planar imaging sequence (repetition time = 2 s; echo time = 29 ms; flip angle = 80°; in plane spatial resolution = 3 × 3 mm^2^; field of view = 240 × 240 mm^2^; slice thickness = 4 mm; number of slices = 32; number of volumes = 240; acquisition time = 8 min). Participants were instructed to lie still with their eyes closed but remaining awake.

#### fMRI preprocessing

The analysis was conducted using pICA as implemented in FSL 4.1.9 (FMRIB Center, Department of Clinical Neurology, University of Oxford, http://www.fmrib.ox.ac.uk/fsl). Data preprocessing consisted of the removal of the first 6 volumes to ensure saturation and adaptation of the subjects to the environment leaving 234 volumes for further analysis, removal of nonbrain structures using Brain Extraction Tool, motion correction using MCFLIRT, high‐pass filtering with a frequency cut‐off at 160 s, low‐pass temporal filtering (5.6 s), spatial smoothing using a Gaussian kernel of full‐width half‐maximum of 5 mm, intensity normalization, and affine linear registration to the MNI152 standard template. Absolute head movement was below 1.5 mm for all subjects.

#### fMRI analysis

pICA identified fifty‐one independent components. We discarded components representing known artifacts, such as motion, high‐frequency noise, or venous pulsation [Beckmann et al., 2005; De Luca et al., 2006], components not located mainly in gray matter, and components not resulting in compact clusters [De Martino et al., 2007]. Finally, components of interest were selected by means of spatial correlation with freely available standard templates of RSNs (http://www.nitrc.org/projects/fcon_1000/) [Biswal et al., 2010], which left us with eighteen anatomically and functionally relevant RSNs.

Subject‐specific statistical maps for the 18 RSNs were created using a dual regression procedure [Filippini et al., 2009] that involves spatial and temporal regression. Then, we estimated differences between the stroke and the healthy control group. The volumetric map of each RSN across subjects was collected into a 4D file to be evaluated for between‐group differences using a nonparametric permutation test (5,000 permutations) [Nichols and Holmes, 2002]. For each RSN, the resulting statistical map was thresholded at *P* = 0.05 and corrected for Family Wise Errors (FWE) employing threshold‐free cluster enhancement (TFCE). Only six RSNs showed significant between‐group difference. Moreover, each of these networks was significantly correlated (*r* > 0.45) with one of the standard template RSNs [Biswal et al., 2010]. We labeled these networks as (1) frontal network (*r* = 0.57), (2) Fronto‐Temporal network (*r* = 0.57); (3) DMN (*r* = 0.55); (4) secondary network (*r* = 0.46); (5) basal ganglia network (*r* = 0.57), and (6) parietal network (*r* = 0.53). Next, we investigated whether these differences were more characteristic to patients with poor cognitive recovery than to patients with good cognitive recovery by means of separate comparison with the healthy control group.

We also entered cognitive scores of test showing significant acute‐to‐subacute difference (see Section “Neuropsychological assessment and grouping criteria regarding cognitive recovery”) into the General Linear Model, Analysis of Covariance (ANCOVA), as covariates of interest to examine whether these cognitive scores showed association with between‐group differences of brain activity at rest. All analyses were thresholded at *P* = 0.05 and corrected for FWE employing TFCE. Anatomical labeling of every result was performed with reference to the Harvard‐Oxford cortical and subcortical structural atlases (http://fsl.fmrib.ox.ac.uk/fsl/fslwiki/Atlases).

## RESULTS

### Sample Characteristics

Demographic and clinical data are given in Table 1. There was no significant difference between stroke patients and healthy controls, except for a higher frequency of diabetes in the stroke group. Since we did not focused on diabetes in this study, we considered it and its nonspecific effect(s) rather as a “confounding factor” regressing it out when performing group analyses. Table 2 contains stroke severity at baseline (NIHSS scale) and characteristics of the ischemic lesions (location, brain hemisphere, volume, and vascular territory). Most patients had lesions in the right hemisphere (15/18) and all infarcts were in the territory irrigated by the MCA with the exception of 2 infarcts located in the PCA territory. Lesions affected one or more of the following regions, ordered by number of subjects affected basal ganglia (*n* = 8), centrum semiovale and temporal lobes (*n* = 7) corona radiata (*n* = 5), insula (*n* = 5), and the frontal lobe (*n* = 5). Comparing the two groups of cognitive recovery, no statistical difference was found in lesion volume (good cognitive recovery: 12.90 cm^3^ [1.13 – 48.23]; poor cognitive recovery: 17.99 cm^3^ [9.80 – 36.00]) (*Z* = −0.446; *P* = 0.656), affected hemisphere or stroke severity at baseline measured by the NIHSS scale (good cognitive recovery: 9.50 ± 6.437; poor cognitive recovery: 10.70 ± 7.027; *t* = −0.7373 (16), *P* = 0.714].

**Table 1:** Demographic and clinical data

	Healthy controls (*n* = 18)	Stroke patients (*n* = 18)		SG (*n* = 8)	SP (*n* = 10)	(SG–SP)
Sociodemographic factors			*P*			*P*
Age (years)	62.61 ± 6.01	63.94 ± 8.26	0.583	61.50 ± 10.14	65.90 ± 6.26	0.274
Women	7 (38.88%)	5 (27.77%)	0.480	2 (25%)	3 (30%)	1.00^3^
Education (years)	7.33 ± 4.1	7.67 ± 4.24	0.812	6.63 ± 3.29	8.50 ± 4.88	0.367
Vocabulary subtest	37.78 ± 7.9	34.61 ± 11.34	0.337	35.25 ± 9.18	34.10 ± 13.29	0.838
Handedness (EHI)	95.56 ± 13.5	97.50 ± 4.29	0.564	97.50 ± 4.63	97.50 ± 4.25	1.000
GDS	2.17 ± 3.42	4.50 ± 4.09	0.072	4.50 ± 3.55	4.50 ± 4.67	1.000
Vascular risk factors						
Hypertension	8 (44.4%)	13 (72.22%)	0.317	5 (62.50%)	8 (80%)	0.608
Dyslipidemia	9 (50%)	10 (55.55%)	0.739	4 (50.00%)	6 (60%)	0.670
Diabetes mellitus	1 (5.55%)	7 (38.88%)	**0.041**	4 (50.00%)	3 (30%)	0.630
Smoking	6 (33.33%)	3 (16.66%)	0.443	1 (12.50%)	2 (20%)	1.000
Alcohol intake	9 (50%)	6 (33.33%)	0.317	1 (12.50%)	5 (50%)	0.152

Independent T‐Test for continuous variables. Chi‐Square test and Fisher's exact test for categorical variables. EHI: Edinburgh Handedness Inventory; GDS: Geriatric Depression Scale; *P*: *P* value for two group comparisons; SG: Stroke patients with good recovery; SP: Stroke patients with poor recovery; Alcohol intake. Diagnosis for a particular vascular risk factor was based in clinical history or use of medication for this particular condition at the time of the clinical assessment.

**Table 2:** Clinical and neuroimaging characteristics of the stroke patients

Patients	Baseline severity (NIHSS)	Infarct side and location	Infarct volume (cm^3^)	Vascular distribution
Stroke patients with good cognitive recovery				
1	1	R. frontal cortex	0.1	MCA_ACA (M2‐M3)
2	2	L. precentral cortex + CR	0.3	MCA (M2‐M3)
3	17	R. basal ganglia	8.2	MCA (M1)
4	9	R. basal ganglia + CR	17.6	MCA (M1)
5	14	R. basal ganglia + insula + CR	36.0	MCA (M1)
6	4	R. occipital cortex + centrum semiovale	53.2	PCA (P2)
7	13	R. insula + temporal and frontal cortex	124.0	MCA‐ACA (M2)
8	16	R. basal ganglia	3.6	MCA
Stroke patients with poor cognitive recovery				
9	5	R. frontal and parietal cortex + premotor cortex + IC	4.6	MCA (M2)
10	22	L. basal ganglia	9.2	MCA (M1)
11	3	L. centrum semiovale	10.0	MCA (M1)
12	7	R. insula + inferior frontal cortex	14.5	MCA (M2)
13	5	R. temporo‐parietal cortex	15.0	MCA (M2‐M3)
14	7	R. temporo‐occipital cortex	20.9	PCA
15	21	R. frontal cortex + lenticulate	24.0	MCA‐ACA (M1)
16	13	R. temporo‐parietal cortex and IC	34.0	MCA (M1)
17	7	R. basal ganglia + CR	42.0	MCA‐ACA (M1)
18	17	R. temporo‐parietal + basal ganglia	175.0	MCA (M1)

Abbreviations: CR: corona radiata; IC: intern capsule; L: left; M1: first segment of the MCA; M2: second segment of the MCA; M3: third segment of the MCA; MCA: middle cerebral artery; NIHSS: National Institute of Health Stroke Scale; P2: second segment of the PCA; PCA: posterior cerebral artery; R: right.

### Neuropsychological Characteristics

Stroke group in general demonstrated a significant acute‐to‐subacute improvement in the following cognitive tests: MMSE, SFT (naming animals in one minute), Boston Naming Test, TMTA, and the GPT (Table 3). We have to emphasize that improvement means increase (score) in the first three and decrease (time to complete) in the last two tests.

**Table 3:** Neuropsychological tests scores at acute and subacute phase for the stroke group

	ACUTEPHASE (within 72 h; *n* = 18)	SUBACUTEPHASE (at 3 months; *n* = 18)	*t* (df)	*P*	*r*
General cognitive function					
MMSE	25.72 ± 3.23	27.22 ± 2.57	−3.040 (17)	**0.007**	**0.35**
Sustained attention					
MoCA subtest	10.11 ± 1.27	10.33 ± 1.28	−0.776(17)	0.449	–
Digit span forward (WAIS‐III)	4.61 ± 1.09	4.72 ± 1.28	−0.46 (17)	0.651	–
Working memory					
Digit span backwards (WAIS‐III)	3.22 ± 1.35	3.44 ± 1.04	−0.940(17)	0.361	–
Premotor functions					
Luria' sequences (/5)	3.61 ± 2.30	4.22 ± 1.66	−1.77(17)	0.094	–
Rhythms subtest (/10)	6.00 ± 2.91	6.83 ± 3.07	−1.567(17)	0.135	–
Interference and inhibitory control (/3)	2.22 ± 1.00	2.50 ± 0.85	−1.426(17)	0.172	–
Verbal fluency					
Letter (P)	7.33 ± 4.25	8.83 ± 4.69	−1.775(17)	0.941	–
Semantic (animals)	10.06 ± 5.23	13.83 ± 4.54	−3.688(17)	**0.002**	**0.44**
Language					
Boston naming test	9.11 ± 3.06	10.83 ± 2.41	−3.511(17)	**0.003**	**0.42**
Understanding (/6)	5.83 ± 0.38	5.94 ± 0.23	−1.458(17)	0.163	–
Psychomotor speed (s)					
Trail making test A (s)	203.89 ± 101.15	107.67 ± 85.48	5.024(17)	**<0.001**	**0.60**
Grooved pegboard test (preferred hand; s)	274.27 ± 74.01	108.39 ± 72.06	7.214(17)	**<0.001**	**0.75**
Visuospatial skills					
Line cancellation test (/36)	30.78 ± 10.38	31.94 ± 7.91	−0.557(17)	0.585	–

Score values are reported as means ± standard deviations for each test (Paired Samples T Test).

Abbreviations: df: degrees of freedom; FBI: frontal behavioral inventory; GDS: geriatric depression scale; MMSE: mini mental state examination; MoCA: montreal cognitive assessment; S‐IQCODE: short informant questionnaire on cognitive decline in the elderly.

*r* = 0.10 (small effect: effect explains 1% of total variance).

*r* = 0.30 (medium effect: effect accounts for 9% of the total variance).

*r* = 0.50 (large effect = effect accounts for 25% of the total variance).

There was no significant difference between the two stroke groups in any cognitive test evaluated in the acute phase. In the subacute phase however, patients with good cognitive recovery performed significantly better in the TMTA (*P* = 0.053) and the number of omissions in the attention subtest (*P* = 0.052) than patients with poor cognitive recovery (data not shown).

### fMRI Analysis

**Figure 2:**
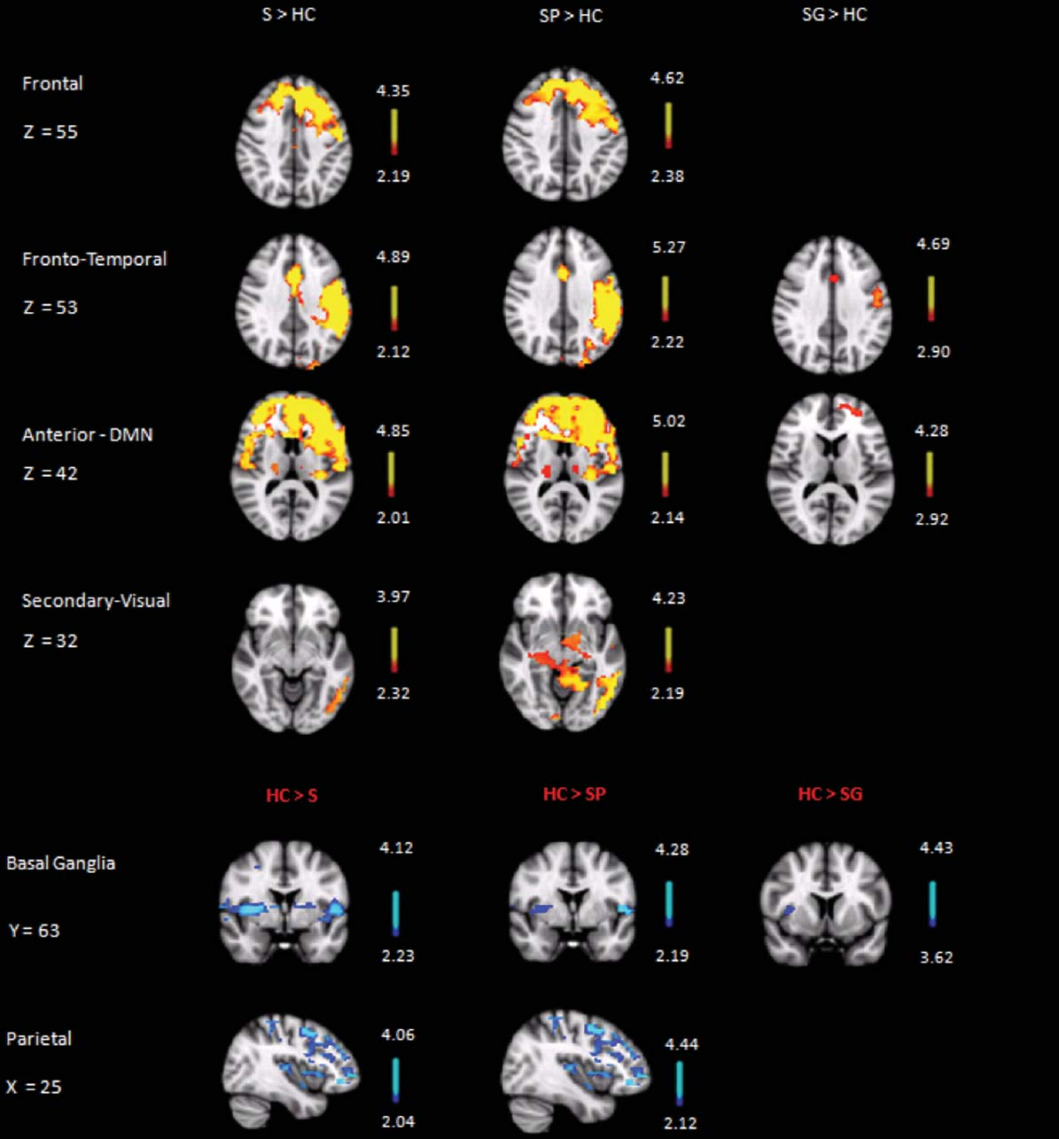
Axial(Frontal, Fronto‐Temporal, DMN and Secondary Visual), coronal (Basal Ganglia), and sagittal (Parietal) slices (MNI template) showing significant between‐group differences in resting activity. HC: healthy control group; S: whole stroke group; SP: stroke patients with poor cognitive recovery; SG: stroke patients with good cognitive recovery. Images are depicted in radiological convention (R‐L).

Compared with the healthy control group, the stroke group showed significant alteration in the following six RSNs: increased brain activity in (1) frontal network; (2) fronto‐temporal network; (3) DMN, (4) secondary network, and decreased brain activity in (5) Basal Ganglia network, and (6) parietal network (Fig. 2, first column)

**Figure 3:**
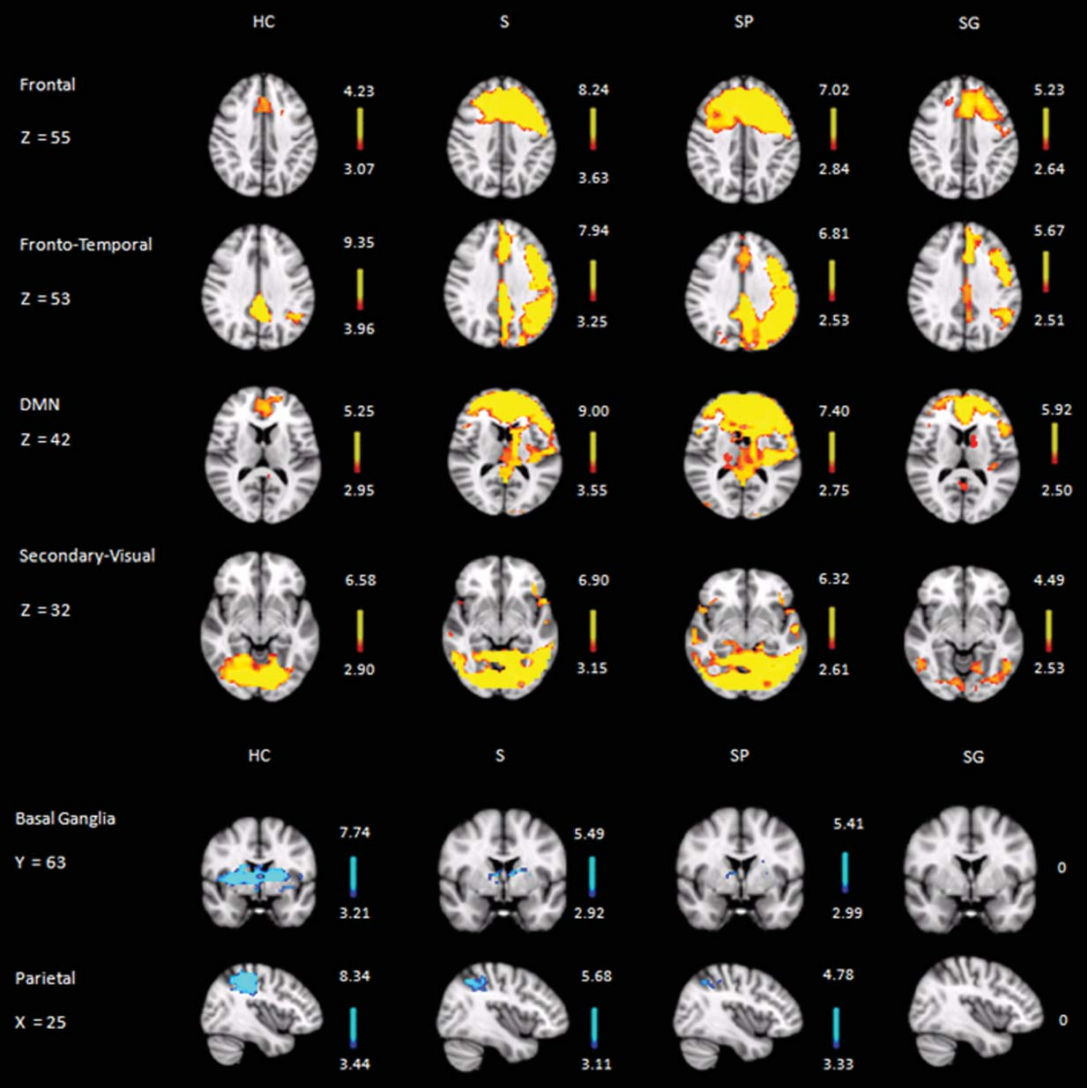
Axial(Frontal, Fronto‐Temporal, DMN and Secondary Visual), coronal (Basal Ganglia), and sagittal (Parietal) slices (MNI template) showing significant resting activity. HC: healthy control group; S: whole stroke group; SP: stroke patients with poor cognitive recovery; SG: stroke patients with good cognitive recovery. Images are depicted in radiological convention (R‐L).

All abovementioned alterations could be detected when comparing patients with poor cognitive recovery separately to healthy control group (Fig. 3, second column). However, patients with good cognitive recovery demonstrated significant increase of activity only in the Fronto‐Temporal and the DMN, as well as significant decrease of activity in the Basal Ganglia network when compared to healthy control group (Fig. 3, third column).

### Relationship Between RSNs Activity and Performance on Cognitive Tests

Whole‐brain ANCOVA Comparing the activity of the significant RSNs in the group of patients with poor cognitive recovery and the healthy control group we found: (a) a lower correlation between Basal Ganglia activity change and SFT score and a higher correlation between Basal Ganglia activity and TMTA time (Table 4), (b) a higher correlation between Frontal activity and SFT score, and (c) lower correlation between Frontal activity and TMTA time (Table 5).

**Table 4:** Clusters of the basal ganglia network showing significant group‐difference in correlations with the scores of the semantic fluency test and the trail making test, part A

Anatomical region	**MNI coordinates**				***P* ≤ 0.05 corrected**	
	X	Y	Z	Voxels mm^3^		
Semantic fluency test						
Healthy control > poor cognitive recovery group						
					*Z*	*P*
R. paracingulate gyrus	21	43	18	3032	4.29	0.002
R. angular gyrus	10	18	30	83	3.61	0.021
L. frontal orbital cortex	28	35	14	20	3.46	0.032
L frontal orbital and frontal operculum cortex	33	38	17	12	3.35	0.043
L. cingulate gyrus, posterior division and precuneus cortex	23	20	21	6	3.33	0.046
Trail making test, part A						
Poor cognitive recovery group > healthy control						
					*Z*	*P*
L. postcentral gyrus	10	24	33	257	3.95	0.007

Note: Correlations between‐group contrasts are cluster corrected for multiple comparison using randomize method (*P* < 0.05TFCE corrected; z‐threshold of 2.3; Critical z for design efficiency calculation set fmri = 5.3).

Abbreviations: L: left; R: right.

Reported *z* values are two‐sided.

**Table 5:** Clusters of the frontal network showing significant group‐difference in correlations with the scores of the semantic fluency test and the trail making test, part A

Anatomical region	MNI coordinates			Voxels (mm^3^)	*P* ≤ 0.05 corrected	
	X	Y	Z			
Semantic fluency test						
Poor cognitive recovery group > healthy control						
					*Z*	*P*
L. angular gyrus	34	18	28	284	3.95	0.007
R. posterior cingulate gyrus and precuneous cortex	20	19	25	178	3.78	0.012
R. angular gyrus	12	16	28	24	3.69	0.016
Trail making test, part A						
Healthy control > poor cognitive recovery group						
L. angular gyrus	35	17	26	1023	3.68	0.017
L. lateral occipital cortex, posterior division	14	11	22	41	3.38	0.04

Note: Correlations between‐group contrasts are cluster corrected for multiple comparisons using randomize method (*P* < 0.05 TFCE corrected; z‐threshold of 2.3; Critical z for design efficiency calculation set fmri = 5.3).

Abbreviations: L: Left; R: Right.

Reported *z* values are two‐sided.

## DISCUSSION

This study aims to identify resting‐state functional connectivity patterns characterizing ischemic stroke in subacute phase and their relations with cognitive recovery. Our pICA analysis identified eighteen relevant components matching the standard RSN reported in healthy subjects [Beckmann et al., 2005]. From these eighteen RSNs, only six showed significant between‐group differences. In comparison with the healthy control group, the stroke group showed increased activity in the Frontal, Fronto‐Parietal, DMN and Secondary Visual networks, and decreased activity in the Parietal and Basal Ganglia networks. These alterations suggest that stroke event affected not only the lesioned hemisphere but the contralesional hemisphere too. Alterations were stronger in stroke patients with poor cognitive recovery, whereas stroke patients with good recovery only showed minimal alterations in three networks (DMN, Fronto‐Temporal and Basal Ganglia networks).

### RSN Connectivity and Motor Recovery in Stroke Populations

Resting‐state studies have already been carried out in stroke populations in relation to motor recovery investigating interhemispheric resting activity as a measure of normal function [Carter et al., 2010; Golestani et al., 2012] and the activity of the ipsilesional primary motor cortex [Park et al., 2011]. They have found that reduced interhemispheric activity at rest is associated with motor deficits [Golestani et al., 2012], recovery of a normal interhemispheric coherence is important for a normal function, and motor impairments are not related to interhemispheric connectivity among attentional‐related areas [Carter et al., 2010]. Furthermore, an increased asymmetry of brain activity at rest is attributed to rearrangements of activation over the bihemispheric sensoriomotor cortex [Park et al., 2011]. These studies were restricted to an a priori selection of specific regions of interest such us the somato‐motor and the attentional network. To supplement these findings, our study focuses on recovery of cognitive functions during the first three months after stroke, which depend on the integration and segregation of several distinct brain networks requiring the study of the brain as a whole.

### RSN Changes in Stroke

There is an on‐going debate in the literature regarding the role of the contralesional hemisphere activity in stroke recovery. Considering the motor function, stroke patients typically show pathologically enhanced neural activity in a number of areas both in the lesioned and in the contralesional hemisphere [Grefkes et al., 2008]. It is pointed out that, early after stroke, the lesioned hemisphere cannot provide transcallosal inhibition, so the other hemisphere becomes hyperactive. These points the research efforts towards two hypotheses: first, that stroke recovery might encompass both degenerative phenomena and mechanisms of plasticity, [Cramer et al., 2008]; and second, that early after stroke contralesional recruitment may be a compensatory adaptation. The second hypothesis explains the multiplicity of deficits following a focal lesion, and the complexity of the neuroplasticity processes that underlie functional brain organization. According to it, in our study, stroke patients with poor cognitive recovery showed increased neural activity at rest in the left (contralesional) hemisphere for the frontal, fronto‐temporal, secondary visual, and the anterior part of DMN.

The brain areas where we found increased activity at rest are related to cognitive functions impaired in our stroke patients, such as executive, attentional, and motor functions (GPT, TMTA, and SFT): the paracingulate cortex involved in top‐down and bottom‐up control to other areas [Allman et al., 2012]; the operculum performs task control [Dosenbach et al., 2008] and switches between the executive control network and the DMN [Seeley et al., 2007]; the anterior insula has been implicated in the salience network, which plays a role in initiation, maintenance and adjustment of attention, and the integrating information [Nelson et al., 2010]; and finally, the frontal pole contributes to inductive, analogical or relational reasoning, as well as prospective memory [Ramnani et al., 2004]. Interestingly, right precuneous cortex in stroke patients with poor cognitive recovery showed both a decreased activity in the Parietal network and an increased activity in the DMN. These findings support the hypothesis that the lesion does not only modify the activity of individual regions but it also affects functional networks as a whole, involving even regions located further from the lesion. Finally, stroke patients showed higher activity at rest in several areas of the secondary visual network. These areas are responsible for visuospatial processing and their lesion may induce neglect [Saalmann et al., 2007]. Most patients with poor cognitive recovery presented neglect in the acute phase so that we hypothesize that recovery during the first three months is related to a compensative over‐activity in contralesional areas. Stroke patients also showed decreased activity in the Basal Ganglia and in the Parietal networks. The former is related to psychomotor speed and attention, while the latter has already been described above.

### RSN Changes as Compensatory Mechanisms

RSN changes by themselves could be interpreted as brain disturbances due to stroke. The fact that they were stronger in stroke patients with poor cognitive recovery also supports this hypothesis. However, a larger portion of brain activity at rest was in the left hemisphere (contralesional in the majority of the patients). This pattern of activity is in agreement with results obtained from stroke recovery research both in animal models and clinical patients showing that widespread changes in activity patterns can even extend to the unaffected hemisphere [Carmichael and Chesselet, 2002; Nelles et al., 1999; Schaechter and Perdue, 2008]. These altered circuits work within the intact contralesional (opposite to stroke) hemisphere [Biernaskie et al., 2005], leading to less lateralized (less crossed) activation.

Most importantly, the magnitude of these changes correlated well with cognitive performance: increased Frontal activity having a positive correlation with cognitive tests, and decreased Basal Ganglia activity having a negative correlation with cognitive tests. The (not significantly) weaker correlations in patients with good cognitive recovery, and the significantly weaker correlation or reverse correlation in healthy controls also support their compensatory nature. In stroke patients with poor cognitive recovery, they seem to have a negative effect on performance probably due to disruption of the interplay between the brain areas. When some of those brain areas are damaged in stroke patients, they compensate these damages via shifting the functional connectivity to favor unaffected brain areas. Therefore, patients demonstrating a larger shift in functional connectivity (i.e., better plasticity) provide a better cognitive performance.

However, these changes seem to play no role in recovery; they actually diminish to allow coming back to “normal” brain activity. That explains their weaker presence in patients with good cognitive recovery. This is in agreement with other studies linking improved recovery with regaining the“normal” brain activity [Dijkhuizen et al., 2012; Ramos‐Cabrer, 2010; van Meer, 2010, 2011].

### Methodological Considerations

Rs‐fMRI is becoming an excellent tool for clinical studies, because it does not impose attentional demands or cognitive burdens on the patient. Although rs‐fMRI has already been employed in other stroke studies, they do not take into account the whole range of brain networks as this study. Moreover, we not only employed a detailed neuropsychological evaluation covering the whole cognitive spectrum in acute stroke; but also investigated how they are associated with recovery. Finally, the study design allows examining how resting‐state brain activity relates to recovery, and whether rs‐fMRI has any predictive value regarding clinically relevant outcome. However, our sample is small due to our strict criteria. This may also decrease the sensitivity, and restrict the generalizability of our preliminary results.

Finally, although we did not reported statistical significant differences regarding the volume of ischemic lesions, their size was heterogeneous. This is a limitation because whereas recovery after a small ischemic lesion may involve preserved peri‐infarct tissue with function similar to the infarcted tissue [Brown et al., 2009; Murphy and Corbett, 2009], for recovery after a large ischemic lesion, tissue with similar function may only be found at more distant sites, such as the premotor cortex (for motor cortex stroke) [Dancause et al., 2005; Frost et al., 2003] or regions in the unaffected contralateral hemisphere [Biernaskie et al., 2005] where structural remodeling has been observed [Takatsuru et al., 2009].

Summarizing, our results confirm our hypotheses and may expand our understanding of brain changes occurring after stroke, as well as stimulate new researches on lesion‐induced network plasticity changes and fMRI biomarkers of recovery/progression not only in stroke but also in vascular cognitive impairment and vascular dementia.

## CONCLUSION

Brain connectivity changes is stroke patients have been already described in task related fMRI studies and in a few resting‐state functional connectivity studies focusing on specific networks. Our less restricted study also demonstrated that these changes affect several brain networks, which not only explains the multiplicity of the deficits following a focal lesion but may also indicate compensatory brain plasticity. As a consequence, they are more pronounced in patients with poor cognitive recovery, whereas patients with good cognitive recovery show “normalization” of these compensatory changes. More importantly, there are strong correlations between functional connectivity changes and cognitive recovery further supporting the relevance of the study of resting‐state functional data.

Our results suggest that resting‐state fMRI provides information for cognitive recovery prognosis and could be a potential biomarker in stroke patients detecting early neural dysfunction and compensatory mechanisms prior to brain atrophy.
